# Mitigation of impedance changes due to electroporation therapy using bursts of high-frequency bipolar pulses

**DOI:** 10.1186/1475-925X-14-S3-S3

**Published:** 2015-08-27

**Authors:** Suyashree P Bhonsle, Christopher B Arena, Daniel C Sweeney, Rafael V Davalos

**Affiliations:** 1 Bioelectromechanical Systems Lab, Virginia Tech-Wake Forest University, School of Biomedical Engineering and Sciences, 330 Kelly Hall (MC0298), Blacksburg, VA. 24061, USA; 2 Laboratory for Therapeutic Directed Energy, Elon University, Department of Physics, Elon, NC. 27244, USA

**Keywords:** Biphasic pulses, Focal ablation, Focal therapy, Electrogenetherapy, Irreversible Electroporation, Electropermeabilization, Electrochemotherapy, Microsecond, Nanosecond, Pulsed Electric Field

## Abstract

**Background:**

For electroporation-based therapies, accurate modeling of the electric field distribution within the target tissue is important for predicting the treatment volume. In response to conventional, unipolar pulses, the electrical impedance of a tissue varies as a function of the local electric field, leading to a redistribution of the field. These dynamic impedance changes, which depend on the tissue type and the applied electric field, need to be quantified a *priori*, making mathematical modeling complicated. Here, it is shown that the impedance changes during high-frequency, bipolar electroporation therapy are reduced, and the electric field distribution can be approximated using the analytical solution to Laplace's equation that is valid for a homogeneous medium of constant conductivity.

**Methods:**

Two methods were used to examine the agreement between the analytical solution to Laplace's equation and the electric fields generated by 100 µs unipolar pulses and bursts of 1 µs bipolar pulses. First, pulses were applied to potato tuber tissue while an infrared camera was used to monitor the temperature distribution in real-time as a corollary to the electric field distribution. The analytical solution was overlaid on the thermal images for a qualitative assessment of the electric fields. Second, potato ablations were performed and the lesion size was measured along the *x*- and *y*-axes. These values were compared to the analytical solution to quantify its ability to predict treatment outcomes. To analyze the dynamic impedance changes due to electroporation at different frequencies, electrical impedance measurements (1 Hz to 1 MHz) were made before and after the treatment of potato tissue.

**Results:**

For high-frequency bipolar burst treatment, the thermal images closely mirrored the constant electric field contours. The potato tissue lesions differed from the analytical solution by 39.7 ± 1.3 % (*x*-axis) and 6.87 ± 6.26 % (*y*-axis) for conventional unipolar pulses, and 15.46 ± 1.37 % (*x*-axis) and 3.63 ± 5.9 % (*y*-axis) for high- frequency bipolar pulses.

**Conclusions:**

The electric field distributions due to high-frequency, bipolar electroporation pulses can be closely approximated with the homogeneous analytical solution. This paves way for modeling fields without prior characterization of non-linear tissue properties, and thereby simplifying electroporation procedures.

## Background

Electroporation is a phenomenon in which transient nanoscale defects referred to as 'pores', form in the cell membrane in response to an externally applied electric field. This increases cell permeability to molecules that before could not pass through the membrane and decreases membrane resistance [1,2]. Electroporation is characterized as reversible when the pores reseal, the membrane recovers after treatment, and the cell survives [3]. This effect is commonly used for introducing chemotherapeutic drugs into tumor cells during electrochemotherapy [4] or for transfer of DNA molecules inside cells during electrogenetherapy [5]. Alternatively, irreversible electroporation (IRE) is characterized by irreversible structural defects, chemical imbalances due to the influx and efflux of ions, and subsequent cell death [3,6]. IRE has shown great promise in the non-thermal ablation of tumors while obviating the need for adjuvant drugs [7,8].

Conventional electroporation-based therapies (EBTs) involve the use of a series of high voltage, unipolar pulses: 8 for electrochemotherapy [4,9] and 80 for IRE [7]. These pulses are on the order of 100 µs in duration and generally delivered at a pulse repetition rate of 1 Hz through electrodes inserted directly into, or adjacent to, the target tissue. For a given tissue type, electrode geometry and spacing, the applied voltage of these pulses generates an electric field distribution in tissue. The resultant local electric field determines the extent of permeabilization--reversible and/or irreversible [10]--or if thermal damage has occurred [7]. However, even if the field distribution is the same, pulse parameters such as pulse number, pulse duration and the temporal mode of delivery of pulses [11] affect the extent of electroporation. Thus for a given treatment protocol, the accurate modeling of the electric field distribution is important for predicting the volume of treated tissue.

For a homogeneous medium with constant electrical properties, the field lines can be obtained using Laplace's equation, which depends only on the applied voltage, electrode size and geometry [12]. However, the electrical properties of biological tissues do not remain constant across the duration of the treatment, as there is an increase in tissue conductivity following electroporation [13-15]. Recent studies utilizing unipolar pulses show that the error in estimation of electroporated area can be reduced significantly by modeling the tissue conductivity as a function of the local electric field magnitude [16-18]. These non-linear properties of biological tissue have to be determined a *priori* [19-21], making treatment planning difficult.

We have previously shown theoretically that bursts of high-frequency, bipolar, square wave pulses on the order of 1 µs produce more homogeneous electrical distributions in heterogeneous tissue geometries [22,23]. The electric currents associated with long pulses that exceed the plasma membrane's characteristic charging time are largely confined to high resistance extracellular spaces prior to the onset of electroporation. However, for short pulses, current can flow through both extracellular and intracellular spaces and the cells experience a uniform electric field, regardless of their packing and morphology [24]. In the present study, we extend the model to include electroporation effects. We hypothesize that the delivery of high-frequency pulses mitigates the effects of conductivity changes due to electroporation compared to conventional unipolar pulses, evoking a more homogeneous tissue response that enables Laplace's equation to more accurately predict the resultant lesion geometry.

**Figure 1:**
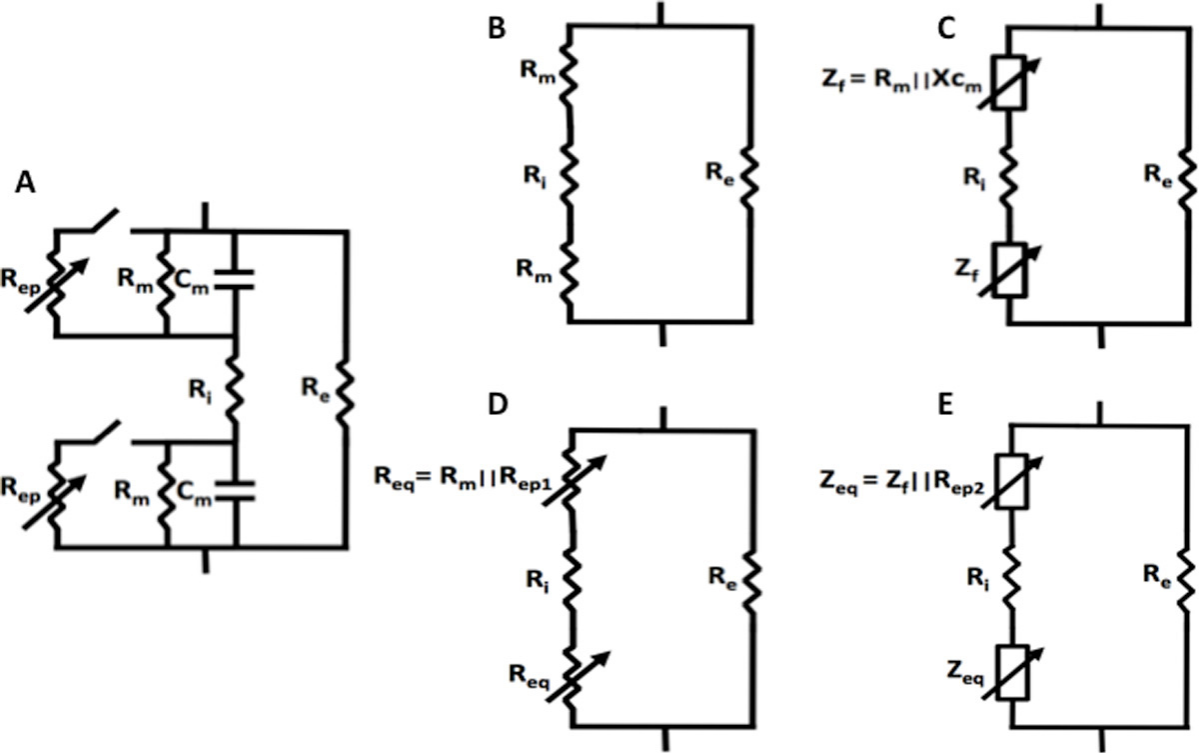
**Functional circuit models representing a single cell exposed to different pulsing waveforms at various stages of electroporation**. A common electrical model was used to represent an intact cell (A). Prior to the onset of electroporation, the membrane capacitance can be considered as an open circuit during conventional unipolar pulse treatment (B), while the contribution of the reactive component to the effective membrane impedance persists for high-frequency bipolar bursts (C). Once membrane permeabilization begins, the equivalent membrane impedance reduces due to electroporation for both the conventional unipolar pulses (D) and high-frequency bipolar bursts (D).

Our hypothesis is based on a common electrical model of a single cell [17,25,26] and its behavior in response to conventional pulses and high frequency, bipolar bursts. Figure 1A shows the intact circuit model of the cell where R_m _and C_m _represent the membrane resistance and capacitance, R_i _the intracellular resistance and R_e _the extracellular resistance. Figure 1B shows the equivalent circuit model when the cell is initially exposed to conventional unipolar pulses, prior to the onset of electroporation. It should be noted that these pulses have a strong DC component, rendering C_m _essentially an open circuit. Additionally, the membrane resistance R_m _is very large (~MΩ), and most current flows through the extracellular resistor R_e_. When high-frequency bipolar bursts are used, the reactance of the capacitor X_Cm_, and therefore the overall initial membrane impedance Z_f _= R_m_||X_Cm _is reduced, because of the significant contribution of the AC components at these frequencies (Figure 1C). As such, the impedance of the cell, when initially exposed to high-frequency pulses, is decreased as compared to that when exposed to conventional pulses.

Once the cellular pores begin to form, more intracellular current pathways are created (R_ep_) resulting in a net reduction in the effective resistance (R_m_||R_ep_). For conventional electroporation pulses, the equivalent impedance is given by R_eq _= R_m_||R_ep1 _(Figure 1D), where C_m _is still considered an open circuit due to the DC component of the pulses. For high-frequency bipolar bursts the overall impedance is modeled by Z_eq _= Z_f _|| R_ep2 _(Figure 1E) because of the relative importance of AC components. While considering a case where the pulse parameters of the each treatment are chosen such that R_ep1 _≈ R_ep2_, it can be seen that, despite the same amount of permeabilization (because R_ep1 _≈ R_ep2_), the overall impedance changes during high-frequency bipolar bursts are reduced because of the lower initial impedance. Ultimately, cell-scale membrane properties play a large role in the bulk tissue properties and for high-frequency pulses; the tissue impedance is less dependent on the extent of electroporation.

To study the effect of conventional unipolar pulses and high-frequency bipolar bursts on electroporation, we performed experiments on potato tissue. Vegetal tissue has been shown to recapitulate the effects of electroporation seen *in vivo*, and lesion boundaries are easily defined due to the marked darkening of the treated areas [16,27]. First, an infrared camera was used to capture the surface temperature of the tissue during treatment. Series of thermal images were used as indirect assessments of the evolution of the electric fields over time. The homogeneous solution was overlaid on one of the temperature distributions to gauge if the dynamic effects of electroporation treatment influenced the resultant electric fields.

Second, potato ablations were made using electroporation pulses and the lesions were analyzed once visibly developed, 24 hours later. The lesion size along the *x*- and *y*-axes were then measured and compared to the analytical solution to assess the predictability of the two pulse protocols with the homogeneous solution. Additionally, electrical impedance measurements (1 Hz to 1 MHz) were recorded immediately before and after the treatments to analyze the dynamic impedance changes caused by the treatments at different frequencies. These data were first analyzed at 1 kHz to compare the extent of electroporation for the two types of treatment. Further, a power spectral analysis was conducted on the delivered unipolar pulse and bipolar burst treatments to assess frequencies that contribute to the impedance of the tissue, for a given type of treatment.

## Methods

### Analytical model of the electric field distribution

For a system of uniform conductivity, the potential distribution and hence the field distribution is given by solving the Laplace equation,

(1) $$\nabla^{2}\Phi =0$$

where  Φ is the electric potential. Specifically, the solution for the electric field distribution from two infinitely long (i.e., 2D) electrodes within an infinite domain can be accurately determined using the following equations [28].

(2) $$|\text{E}\left(\text{x},\text{y}\right)|=\text{C}\cdot \sqrt{{\left(\frac{\text{x}\prime_{\text{A}}-\text{x}}{{\left(\text{x}\prime_{\text{A}}-\text{x}\right)}^{2}+{\left(\text{y}\prime_{\text{A}}-\text{y}\right)}^{2}}-\frac{\text{x}\prime_{\text{B}}-\text{x}}{{\left(\text{x}\prime_{\text{B}}-\text{x}\right)}^{2}+{\left(\text{y}\prime_{\text{B}}-\text{y}\right)}^{2}}\right)}^{2}+{\left(\frac{\text{y}\prime_{\text{A}}-\text{y}}{{\left(\text{x}\prime_{\text{A}}-\text{x}\right)}^{2}+{\left(\text{y}\prime_{\text{A}}-\text{y}\right)}^{2}}-\frac{\text{y}\prime_{\text{B}}-\text{y}}{{\left(\text{x}\prime_{\text{B}}-\text{x}\right)}^{2}+{\left(\text{y}\prime_{\text{B}}-\text{y}\right)}^{2}}\right)}^{2}}$$

where $C=\frac{v_{AB}}{2\cdot \text{log}\frac{d_{AB}+\sqrt{{d_{AB}}^{2}-4\cdot a^{2}}}{2\cdot a}}$, $e=\frac{d_{AB}}{2}-\sqrt{{\left(\frac{d_{AB}}{2}\right)}^{2}-a^{2}}$, $x\prime_{A}=x_{A}+|e|\cdot \text{cos}\theta_{1}$$\theta_{1}=\text{arctan}\left(\frac{y_{B}-y_{A}}{x_{B}-x_{A}}\right)$, $x\prime_{B}=x_{B}+|e|\cdot \text{cos}\theta_{2}$, $\theta_{2}=\text{arctan}\left(\frac{y_{A}-y_{B}}{x_{A}-x_{B}}\right)$, $y\prime_{A}=y_{A}+|e|\cdot \text{sin}\theta_{1}$, $y\prime_{B}=y_{B}+|e|\cdot \text{sin}\theta_{2}$, V_AB _is the voltage, d_AB _is the distance between the electrodes, a is the electrode diameter, x_A_, y_A _and x_B_, y_B _are the geometrical centers and $x\prime_{A}$, $y\prime_{A}$ and $x\prime_{B}$, $y\prime_{B}$ are the mathematical equivalents. The analytical solution to Laplace's equation represents an idealized electric field distribution for a homogeneous medium of constant-impedance. This solution was plotted in a script in MATLAB 2014b (MathWorks, Inc., Natick, MA, US).

### Thermal imaging of vegetative tissue

**Figure 2:**
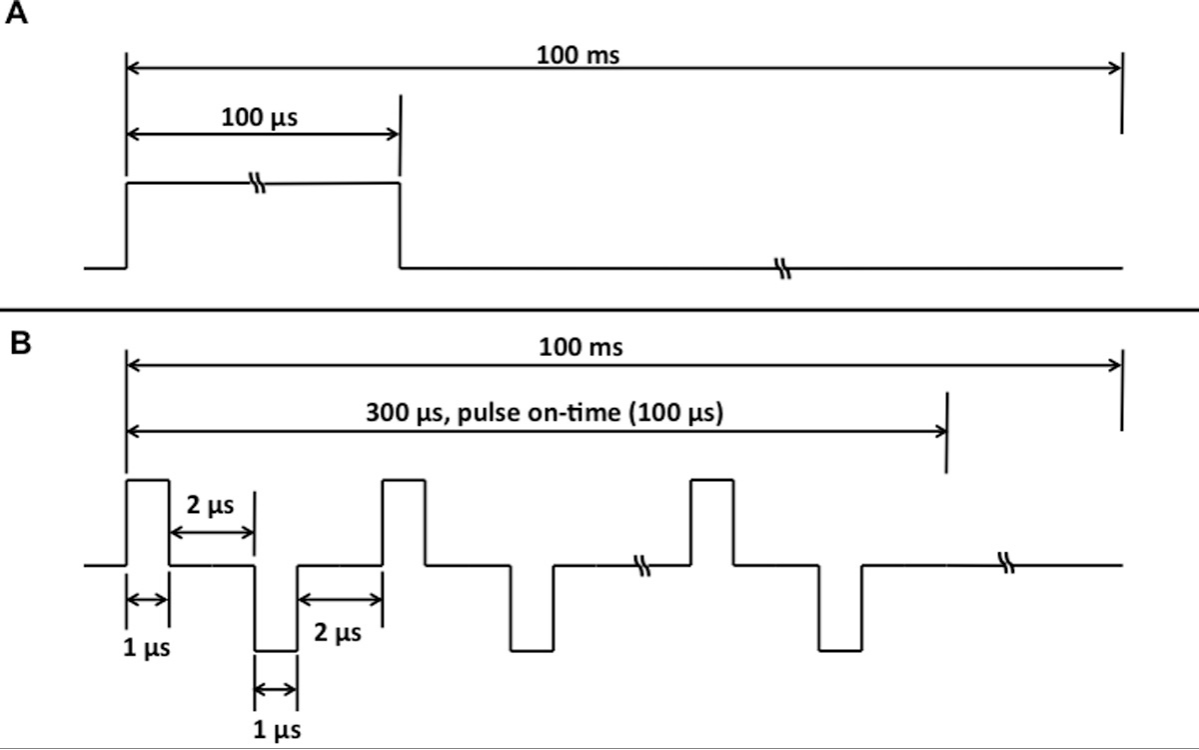
**Pulse waveforms delivered to the potato tissue during pulsing in their idealized form**. For unipolar pulse treatment, pulse widths of 100 µs were delivered at a pulse repetition frequency of 10 Hz (100 ms duty cycle) for 80 pulses (A). For high-frequency bipolar bursts, 50 bipolar pulses (1 µs positive, 2 µs delay, 1 µs negative, 2 µs delay) were delivered at a burst repetition frequency of 10 Hz (100 ms duty cycle) for 80 bursts.

Uniformly sliced, 1-cm thick, russet potato tissue samples were pulsed with conventional unipolar pulses, 100 µs duration (Figure 2A) and high-frequency, bipolar bursts, 1 µs pulse duration, 100 µs on-time (Figure 2B). Two 1-mm diameter electrodes with 1-cm spacing and 1-cm exposure length were used for the study. The thermal images were captured using an Infrared (IR) camera (ICI 7320, Infrared Cameras Inc, TX, US) at a frame rate of 10 images per second, during pulsing. The amplitude of the pulses delivered was chosen to be 800 V to enable the thermal changes to be visualized by the IR camera yet remain below the threshold for thermal damage [29]. For the first 40 unipolar pulses or bipolar bursts (also 40 frames), no significant increase in temperature (less than 1°C) was observed and, therefore, only the later 40 frames were used for the analysis. The images were imported into ImageJ (National Institute of Health, MA, US) and a video (Additional file 1) was generated of the temperature evolution in real-time over the last 40 frames.

Unipolar pulses were applied using the BTX ECM 830 electroporation system (Harvard Apparatus, Holliston, MA, US), and bipolar bursts were delivered using a custom-made pulse amplifier (Applied Energetics, Inc., Tucson, AZ, US) that amplified high-frequency waveforms from an arbitrary function generator (AFG3021C, Tektronix Inc., Beaverton, OR, US). More details of this system are provided in [30,31]. The unipolar protocol consisted of 80, 100 µs pulses delivered at a pulse repetition frequency of 10 Hz (100 ms total time). The bipolar bursts consisted of a train of 100, 1 µs duration pulses of alternating polarity, with a delay of 2 µs programmed between each pulse to protect the MOSFET based pulse generator, as shown in Figure 2B. A total of 80 such bursts were delivered at a burst repetition frequency of 10 Hz (100 ms total time). In this protocol, the voltage and pulse on-time during both types of treatment was equivalent.

The repetition rates for high-frequency pulses were delivered at rates higher than the traditional 1 Hz so that the heat dissipated between pulses would be small. This would cause the increase in temperature be proportional to the heating term throughout treatment and not just during the on time of pulses. The heating is a consequence of the electrical power dissipated in the tissue, which is given by Joule's first law Q = σ|**E**|^2^, where Q is the energy, σ is the electrical conductivity and **E **is the electric field intensity. For homogeneous materials with low thermal conductivity (potato tissue thermal conductivity between 0.545 to 0.957 W/m°C [32]) and constant electrical conductivity, the increase in temperature is proportional to the square of the magnitude of the electric field. Accordingly, we can expect the resultant temperature distributions to closely follow with the homogeneous analytical solution of the electric fields when the electrical impedance is constant.

While, the underlying electric field distribution was indirectly evaluated by monitoring the temperature distribution, the purpose of this study was not to obtain a one-to-one mapping of the temperature and electric fields, but rather to provide a qualitative analysis of whether the distributions were a consequence of unchanging electrical properties. The homogeneous electric field distribution was obtained from Laplace's solution at V_AB _= 800V, d_AB _= 1 cm and a = 1 mm (i.e. applied voltage, electrode spacing and dimensions that were used to conduct the IR experiments). The electric field distributions were overlaid on the thermal images with electrodes as reference points. Only the smallest electric contours that encompassed each temperature contour were chosen for the overlay. The alignment of the electric and temperature contours was evaluated spatially to gauge if the temperature maps were a consequence of static impedance.

### Vegetative tissue ablation

Russet potato slices of 5-mm thickness were ablated to analyze the lesions created by unipolar pulses and bipolar bursts. The pulse generators, electrode dimensions and spacing, and number of unipolar pulses or bipolar bursts used for treatments were the same as above. In this set of experiments, the pulse/burst repetition frequency was 1 Hz, which is standard for IRE protocols. The peak amplitude of the bipolar bursts was 1020 V whereas that of the unipolar pulses was 400 V. These amplitudes were chosen so that the two therapies led to comparable lesion sizes [31].

In potato tuber tissue, the irreversibly electroporated area is distinctively darkened 5 hours after electroporation due to an accelerated oxidation of chemical constituents caused by a decompartmentalization of certain enzymes and substrates [33] that occurs at cell lysis caused by electroporation [16]. Five trials (*n *= 5) of each type of treatment were performed and the samples were covered and placed on a bench top post-treatment. Images of the lesions were obtained 24 hours after electroporation using HDSLR camera Nikon D3200 (Nikon Corporation, Chiyoda, TYO, JP) in a well-illuminated area at a distance of ~0.5 m from the sample. The white balance was manually pre-set using a sheet of white paper, prior to taking the images.

The images obtained from our study were first converted to 8-bit grayscale images. The pixel values in the fifth row and fifth column (arbitrarily chosen background rows/columns) were averaged to provide an estimate of the background values in each image. It was observed in a prior study [16] that the irreversibly electroporated area in potato tissue was 30% darker or more than the surrounding tissue. Accordingly, a thresholding operation was performed such that the pixels that were less than 30% of the calculated background pixel value were set to 0 (black), while the remaining were set to 255 (white). Considering only the contiguous areas, the lesion sizes were measured along the *x- *and *y*-axes. These lesions were compared to the smallest analytical solution contour that included the entire contiguous thresholded region (defined as ablation threshold).

### Impedance sweep and power spectral analysis

Electrical impedance measurements for were made prior to, and immediately after, the potato tissue ablations (*n *= 5) using a Gamry Reference 600 potentiostat/galvanostat (Gamry, Warminster, PA, US) in the frequency range of 1 Hz to 1 MHz at ten points per decade to analyze the dynamic impedance changes caused by the ablations at different frequencies. The time between treatment and the impedance sweep was always between 17 and 23 seconds. These sweeps were qualitatively analyzed to determine how the impedance before and after electroporation is impacted when measuring at different frequencies. Further, a power spectral analysis was conducted on the input unipolar pulses and bipolar burst trains in a script in MATLAB to assess frequencies that contribute to the impedance of the tissue, for a given type of treatment.

## Results and discussion

### Thermal imaging allows for indirect visualization of electric field evolution

**Figure 3:**
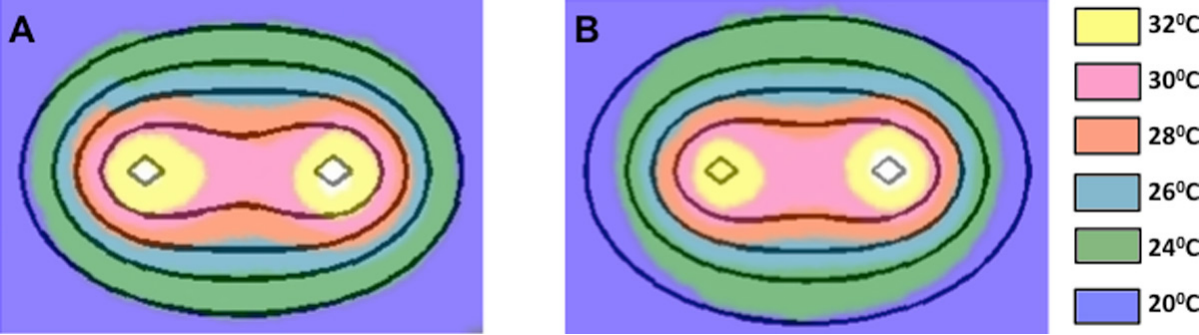
**Representative thermal images overlaid with the corresponding electrical field contours from the analytical solution of the Laplace's equation, using the electrode insertions as the reference points for the mapping**. Thermal image of high-frequency bipolar treatment was overlaid with the electric field analytical solution at 3000 V/cm, 600 V/cm, 350 V/cm, 240 V/cm, and 160 V/cm going outward from electrode (A). Thermal image of conventional unipolar treatment was overlaid with the electric field analytical solution at 3000 V/cm, 500 V/cm, 300 V/cm, 200 V/cm, and 120 V/cm going outward from electrode (B).

Images obtained from the thermal camera provide a qualitative assessment of the electric field distribution in potato tissue. When the impedance of the tissue is static (does not depend on the extent of electroporation), the increase in temperature is directly proportional to the square of the field. Accordingly, the measured temperature contours closely follow the electric field contours predicted by Laplace's equation. The electric field contours have been overlaid on the thermal images (Figure 3) using the electrode insertions as the reference points for the mapping. When using high-frequency bipolar bursts, the temperature distributions were more closely emulated by the electric field distributions (Figure 3A) than the unipolar pulses (Figure 3B). This indicates that the dynamic changes in tissue properties due to electroporation have a minimal impact on the evolution of the electric field distribution when high-frequency bursts are applied. When applying conventional unipolar pulses, larger geometric disparities were observed between the measured temperature and the Laplace electric field contours (Figure 3B). Under these conditions, the effects of electroporation greatly influence the electric field distribution (as indicated indirectly by the altered temperature distribution). Specifically, the temperature distribution becomes compressed along the axis joining the electrodes. This is characteristic of lesions observed clinically in several tissue types, including kidney [34].

### Electric field distributions at high frequencies can be predicted by Laplace's equation

**Figure 4:**
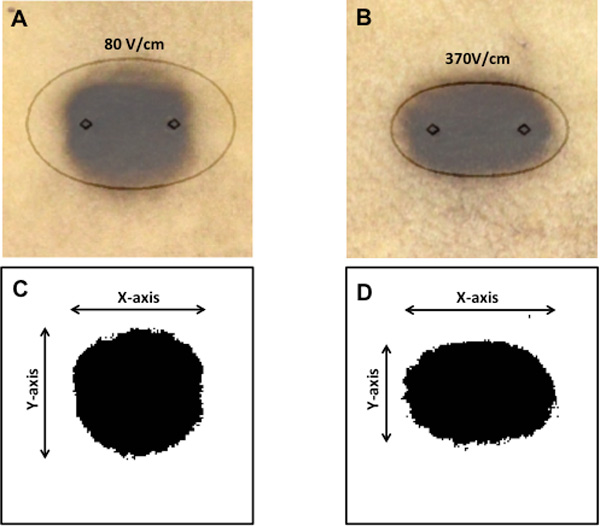
**Laplace's equation more accurately represents the lesion geometry generated by high-frequency bipolar bursts**. Lesions obtained due to conventional unipolar treatment with 80 pulses of 400 V, 100 µs duration, and pulse repetition rate of 1 Hz were overlaid with Laplace's solution contour plotted for 80 V/cm electric field intensity (A). Lesions obtained due to high-frequency bipolar burst treatment with 80 bursts of 1020 V, 100 µs on time, and pulse repetition rate of 1 Hz were overlaid with Laplace's solution contour plotted for 350 V/cm electric field intensity (B). Thresholded lesion geometry from image A (C) and image B (D).

Images of potato ablations were compared to the electric field contours determined by the analytical solution as a quantitative measure of treatment predictability (Figure 4). The mean lesion size ± standard deviation in the *x- *and *y-*direction for conventional unipolar pulses and high- frequency bipolar burst treatment are shown in Table 1. For unipolar pulses, the lesion size was 1.49 ± 0.03 cm in the *x*-direction and 1.40 ± 0.07 cm in the *y*-direction. For bipolar bursts, the lesion size was 1.65 ± 0.03 cm in the *x*-direction and 1.09 ± 0.06 cm in the *y*-direction. The ablation thresholds were defined as the electric field contour enveloping the lesions. This threshold was 80 V/cm for unipolar pulses (Figure 4A) and 350 V/cm for the high-frequency bipolar bursts (Figure 4B). For unipolar pulse treatment, the measured size of the smallest electric field contour that encompassed the entire contiguous lesion was 2.47 cm in the *x-*direction and 1.50 cm in the *y*-direction, whereas for high-frequency burst treatment was 1.95 cm in the *x-*direction and 1.05 cm in the *y*-direction

**Table 1:** Statistical Data Potato Lesions.

Measurements		Conventional Unipolar Pulse Treatment Sample Size n = 5	High-Frequency Burst Treatment Sample Size n = 5
**Pulse Parameters**		**100 µs, 80 pulses, 1 Hz**	**1 µs, 100 µs on-time, 80 bursts, 1 Hz**

	Size of lesion in X-axis (cm)	1.49 ± 0.03	1.65 ± 0.03
	
	Size of lesion in Y-axis (cm)	1.40 ± 0.07	1.09 ± 0.06
Lesion Dimensions mean ± std deviation			
	Percent difference from ablation threshold in X-axis (%)	39.70 ± 1.30	15.46 ± 1.37
	
	Percent difference from ablation threshold in Y-axis (%)	6.87 ± 6.26	3.63 ± 5.90

For unipolar pulses, the percent differences in lesion sizes from the threshold predicted by the analytical solution were found to be 39.70 ± 1.30 % in *x-*direction and 6.87 ± 6.26 % in the *y-*direction. For bipolar bursts, the percent differences in lesion sizes from the threshold were 15.46 ± 1.37 % in *x-*direction and 3.63 ± 5.90 % in the *y-*direction. These results show that Laplace's equation can closely predict the electric field threshold of ablation for high-frequency bipolar bursts.

### Higher frequencies motivate greater consistency in impedance during electroporation

**Figure 5:**
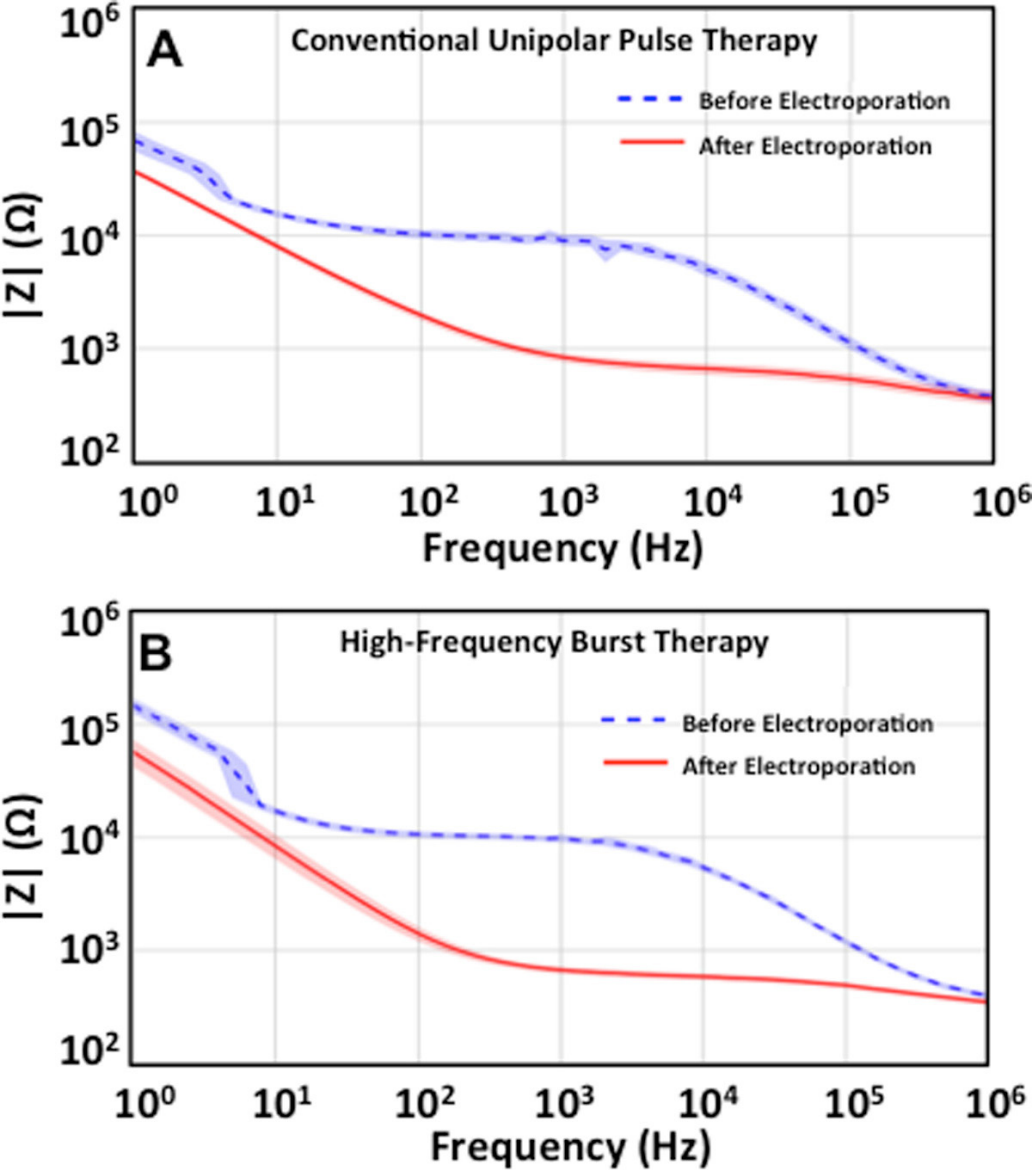
**Frequency spectrum (95% confidence interval around mean) obtained prior to and after treatment of potato tissue with conventional unipolar pulses and high-frequency bipolar bursts showing similar shifts in impedance spectra**. Blue dotted line indicates the frequency spectrum obtained prior to treatment with unipolar pulses. Solid red line indicates frequency spectrum obtain after treatment of tissue with unipolar pulses (A). Blue dotted line indicates the frequency spectrum obtained prior to treatment with high-frequency bipolar bursts. Solid red line indicates frequency spectrum obtain after treatment of tissue with high-frequency bipolar bursts (B).

The impedance sweeps before and after electroporation with unipolar pulses and bipolar bursts are shown in Figure 5A with 95% confidence interval around the mean. It is important to note that the impedance measurements at lower frequencies are dominated by electrode-tissue capacitance for a 2-electrode configuration. However, for frequencies above 1 kHz, the effect of this capacitance is reduced and useful information regarding the tissue can be obtained [35]. Accordingly, the bioimpedance measurements, for both types of treatments, above 1 kHz qualitatively show that the effects of impedance changes due to electroporation are mitigated when tissue is exposed to non-permabilizing higher frequencies.

**Figure 6:**
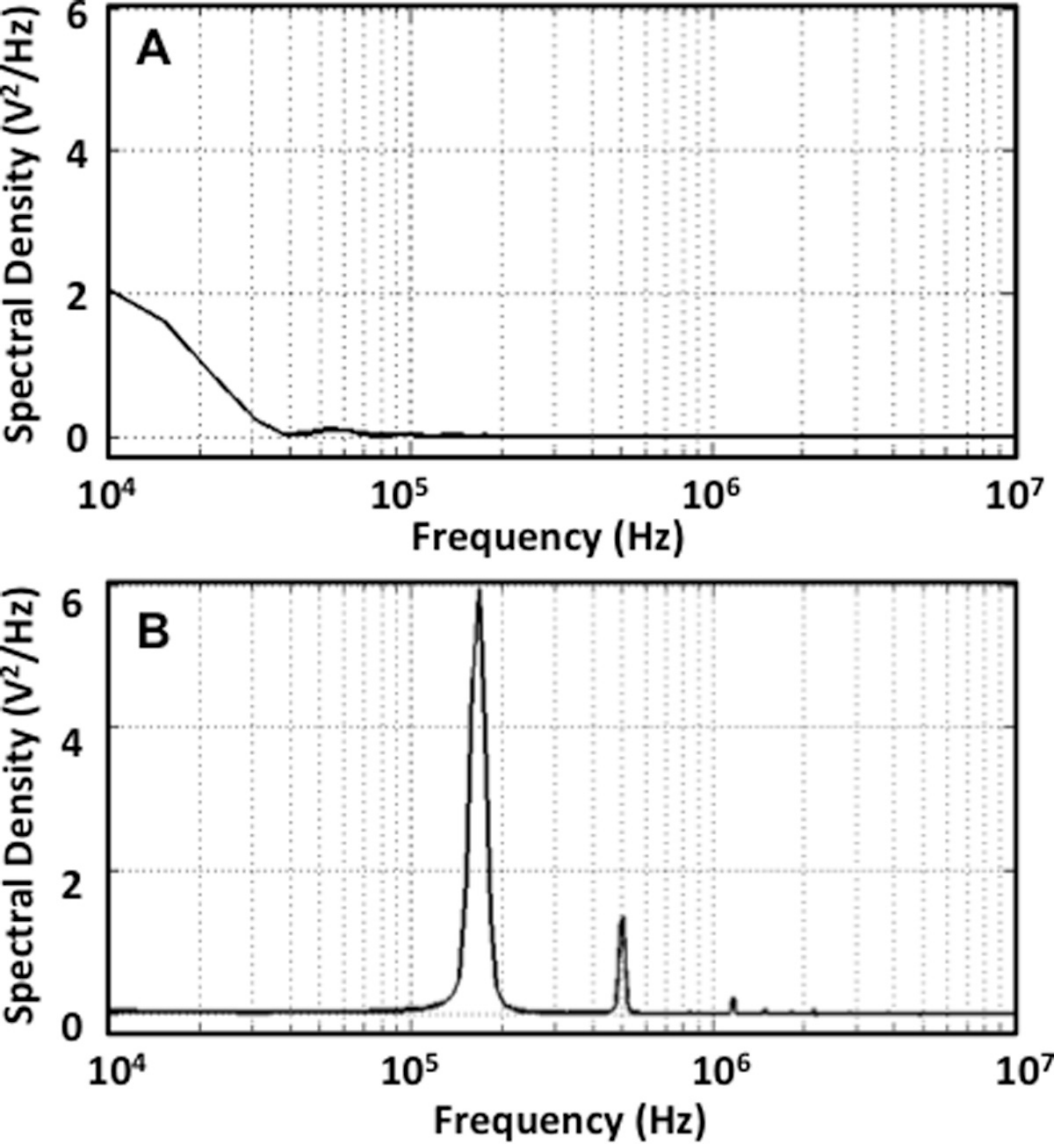
**A power spectral analysis of the input waveforms - conventional unipolar pulse (A) and a high-frequency bipolar burst (B) was obtained**.

Furthermore, a power spectral analysis of the two waveforms - unipolar pulse and bipolar burst (Figure 6) reveals that they contain different frequency content. For the input unipolar pulse (Figure 6A) delivered to the potato tissue during ablations, most amount of power of the signal is concentrated between 10 kHz and 40 kHz. Whereas the spectral density of a high-frequency bipolar burst is concentrated around 167.8 kHz i.e. reciprocal of the total pulse period, where total pulse period = 2 × 1 µs (pulse period) + 2 × 2 µs (interphase delay), and 500 kHz i.e. 1/(2 × 1 µs (pulse period)). A possible explanation of why the potato tuber appears more homogeneous when exposed to high-frequency bursts is that the higher frequency spectral content of the pulses reduces the effects of impedance changes during treatment. The unipolar pulses, on the other hand, contain lower frequency content wherein the effects of changes in impedance are still visible. This suggestion is consistent with the bioimpedance measurements made at non-permeabilizing voltages.

The effect of macroscopic homogeneity in tissue during electroporation treatment could be enhanced as the total pulse period is reduced. However, as the pulse frequency increases (i.e. pulse duration decreases) the electric field thresholds of permeabilization increase [31,36] leading to higher costs and necessary safety precautions associated with the pulse generator technology [37,38]. Furthermore, the pores created by nanosecond pulses result in decreased permeability to large molecules [39]. Therefore, depending on the EBT application, a tradeoff should exist between the pulse length and the accuracy of prediction of electric fields using the homogeneous solution.

### Future work

For EBTs, currently there are only two methods of evaluating electric field distributions in tissue *in situ*, electrical impedance tomography (EIT) [40] and magnetic resonance electrical impedance tomography (MREIT) [41]. These techniques require sophisticated equipment and complex algorithms that would be valuable to develop for a clinical setting. However, for laboratory *ex vivo *experiments (no blood perfusion and low thermal conductivity) when such systems are not readily available, the proposed method of thermal imaging could serve as a useful and simple alternate to assess the electric field distributions. To accurately determine the value of the electric field distributions, a one-on-one mapping of the temperature and electric fields would be necessary and must be further optimized. For conventional unipolar pulses, dynamic impedance changes during electroporation need to be taken into consideration for the mapping. It should be noted that since the thermal camera can only capture the surface temperatures, the electrode placement and tissue surface must be uniform throughout the sample.

Prior theoretical studies have shown that high-frequency pulses have the potential to mitigate tissue heterogeneities [22]. But, future experimental work needs to be conducted to validate these models in heterogeneous environments. Such a study would be of paramount importance as it would show that tissue-to-tissue variability could potentially be reduced during electroporation therapy, with one solution fitting all tissues, for a given geometry and electrode. Furthermore, studies on IRE of liver tumors have shown that treatments in heterogeneous environments contribute to local recurrences of tumors due to redistribution of electric fields [42,43]. Eliminating the need to map the electrical properties by mitigating effects of impedance changes, as shown in this work as well as reducing effective tissue inhomogeneity could improve treatment outcome of EBTs.

Here, proof-of-concept experiments were performed in order to investigate the potential of high-frequency bursts to simplify treatment planning. We experimentally determined that the non-linear change in impedance during electroporation is reduced using high frequency pulses and electric field distribution can be determined from Laplace's equation alone. The implications of this study are that electroporation effects would not need to be quantified a *priori* making prediction of treatment outcome simpler.

## Conclusion

In this study, we examined the predictability of high-frequency, bipolar bursts with the analytical solution to Laplace's equation, which is independent of the electrical properties of the tissue. We analyzed the electric fields experimentally in potato tissue during electroporation. The results indicate that the impedance changes during electroporation treatment are mitigated when using high-frequency pulses and, consequently, the resultant electric field can be modeled using the analytical solution. When implemented clinically, high frequency waveforms have the potential to produce more predictable outcomes due to the mitigation of electroporation dependent field effects.

## Abbreviations

irreversible electroporation (IRE), electroporation-based therapy (EBT), electrical impedance tomography (EIT), magnetic resonance electrical impedance tomography (MREIT)

## Competing interests

CBA and RVD have patents pending on this technology.

## Authors' contributions

SPB, CBA and RVD designed and performed all experiments. DCS assisted in impedance and power spectral analysis. All authors read and approved the final manuscript.

## Supplementary Material

Additional file 1 **Thermal video of conventional unipolar and high-frequency burst treatment during pulsing potato tuber tissue**. Click here for file

## Declarations

The publication costs for this article were funded by Virginia Tech's Open Access Subvention Fund. The research costs were supported in part by the NSF and CIT under Awards CAREER CBET-1055913, CIT CRCF MF13-034-LS and NSF IIP 1346343.

This article has been published as part of *BioMedical Engineering OnLine *Volume 14 Supplement 3, 2015: Select articles from the 6th European Conference of the International Federation for Medical and Biological Engineering (MBEC 2014). The full contents of the supplement are available online at http://www.biomedical-engineering-online.com/supplements/14/S3.
